# Direct regulation of E-cadherin by targeted histone methylation of TALE-SET fusion protein in cancer cells

**DOI:** 10.18632/oncotarget.4340

**Published:** 2015-06-19

**Authors:** Hyun-Soo Cho, Jeong Gu Kang, Jae-Hye Lee, Jeong-Ju Lee, Seong Kook Jeon, Jeong-Heon Ko, Dae-Soo Kim, Kun-Hyang Park, Yong-Sam Kim, Nam-Soon Kim

**Affiliations:** ^1^ Genomics Structure Research Center, Korea Research Institute of Bioscience and Biotechnology, Daejeon, 305-333, Republic of Korea; ^2^ Human Derived Material Center, Korea Research Institute of Bioscience and Biotechnology, Daejeon, 305-333, Republic of Korea; ^3^ Aging Intervention Research Center, Korea Research Institute of Bioscience and Biotechnology, Daejeon, 305-333, Republic of Korea; ^4^ Department of Functional Genomics, Korea University of Science and Technology, Daejeon, 305-333, Republic of Korea

**Keywords:** TALEN, histone methylation, migration, cancer

## Abstract

TALE-nuclease chimeras (TALENs) can bind to and cleave specific genomic loci and, are used to engineer gene knockouts and additions. Recently, instead of using the FokI domain, epigenetically active domains, such as TET1 and LSD1, have been combined with TAL effector domains to regulate targeted gene expression via DNA and histone demethylation. However, studies of histone methylation in the TALE system have not been performed. Therefore, in this study, we established a novel targeted regulation system with a TAL effector domain and a histone methylation domain. To construct a TALE-methylation fusion protein, we combined a TAL effector domain containing an E-Box region to act as a Snail binding site and the SET domain of EHMT 2 to allow for histone methylation. The constructed TALE-SET module (TSET) repressed the expression of E-cadherin via by increasing H3K9 dimethylation. Moreover, the cells that overexpressed TSET showed increased cell migration and invasion. This is the first phenotype-based study of targeted histone methylation by the TALE module, and this new system can be applied in new cancer therapies to reduce side effects.

## INTRODUCTION

Transcription activator-like (TAL) effector nucleases (TALENs) can introduce genetic modifications such as gene knockouts and additions, by selective genomic cleavage [1, 2]. Previously, a TALEN-based knockout library for human microRNAs was constructed, and targeted gene knockout was performed in chickens, mice and zebrafish [3–6]. In addition, the TALEN system was applied for targeted regulation in cancer and for the pluripotent stem cell (iPS) cells [7, 8].

Several papers have reported that epigenetically active domains, such as TET1 and LSD1, combined with TAL effector domains can successfully regulate targeted gene expression. TALE-TET1 directly activated the expression of KLF4, RHOXF2 and HBB via targeted DNA demethylation. Furthermore, TALE-LSD1 was capable of demethylating enhancer-associated chromatin modifications [9, 10]. However, studies of histone methylation in the TALE system have not been performed.

EMT (Epithelial-Mesenchymal Transition) is a critical phenotypic change during tumor metastasis. In the EMT process, epithelial cells gain characteristics of fibroblasts and show increased cell motility and decreased intercellular adhesion. In addition, EMT markers (E-cadherin, cytokeratin and claudin) are reduced, but MET markers (N-cadherin and vimentin) are increased [11–13]. Recently, *Dong et al*. reported that a G9a-Snail complex decreased E-cadherin expression by increasing H3K9 dimethylation. Di- and trimethylation of H3K9 provides a binding site for chromodomain-containing proteins of the heterochromatin protein 1 family; this binding leads to gene repression via changes in heterochromatin structure [14, 15], and this epigenetic regulation by G9a decreases E-cadherin expression and, subsequently, increases cell migration in breast cancer cell lines [16]. In addition, many histone methyltransferases and demethylases are involved in cancer growth and metastasis [17–22].

In this study, we established a new, targeted regulation system with a TAL effector domain and a histone methylation domain and constructed a TALE-SET module (TSET) for targeted regulation of E-cadherin via epigenetic regulation of the histone code in cancer cells. We found that expression of E-cadherin was repressed and cell migration and invasion were increased by TSET. Therefore, these results imply that targeted methylation by TSET is a new method for controlling side effects and for gene regulation in cancer therapy.

## RESULTS

### Construction of TSET for inhibition of E-cadherin

**Figure 1:**
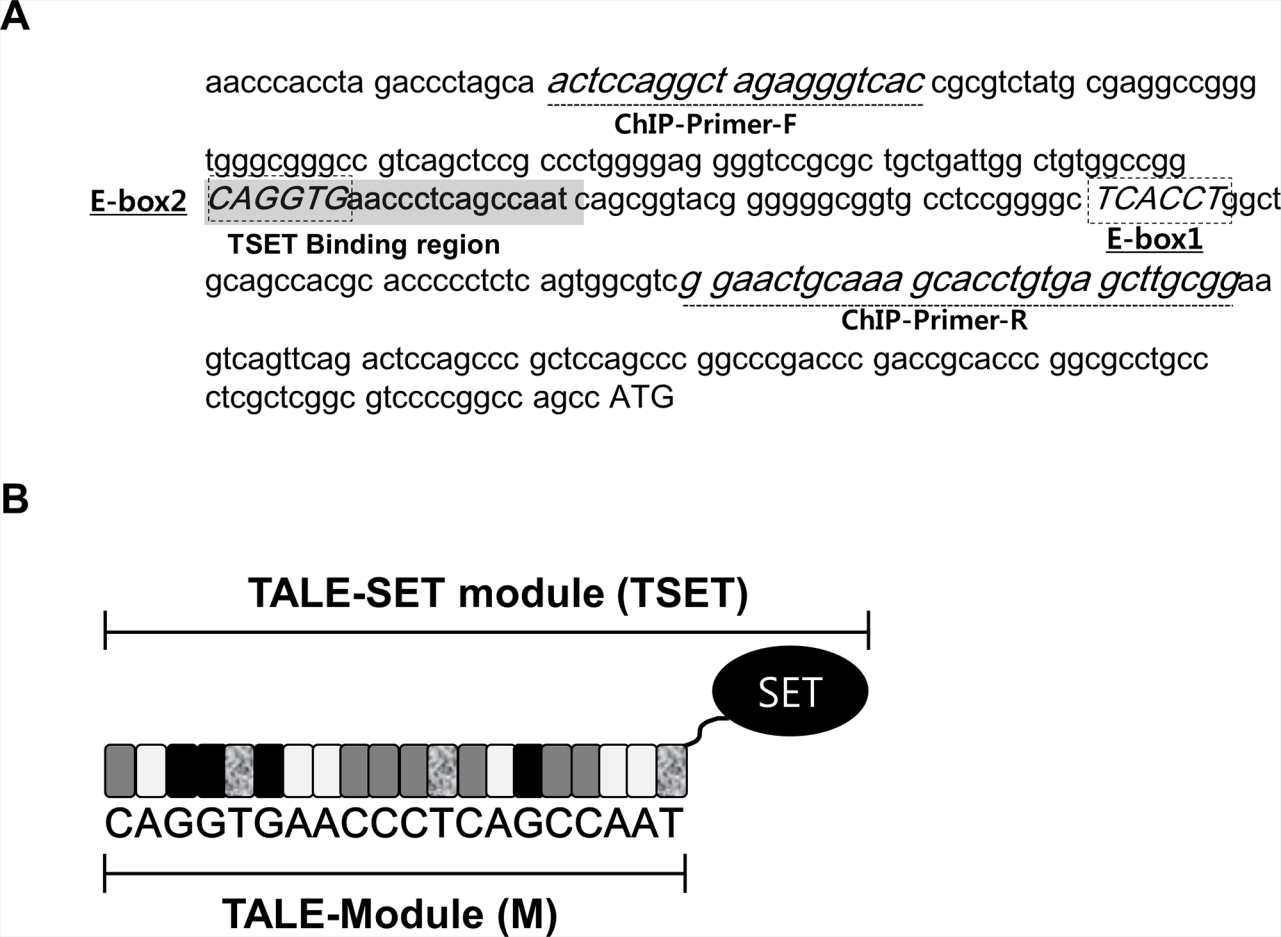
Construction of TSET (TALE-SET module) **A.** Sequence of promoter region of E-cadherin and target site in TALE module. Dot-box (E-Box1/2 sequence; Snail binding site), Gray highlight (TSET binding region), dot-line (ChIP-Primers), ATG (Start codon). **B.** Diagrammatic sketch of TSET. SET (SET domain), Gray box (C), White box (A), Black box (G), dot box (T).

The expression of E-cadherin was repressed by a G9a-Snail complex via epigenetic modifications of the gene's promoter region [16]. In this paper, to establish a targeted histone methylation system with TAL effector repeat domains, we focused on E-cadherin regulation by the G9a-Snail complex. Because G9a can bind to DNA when complexed with Snail and can methylate histone H3K9, we designed a TAL effector domain containing the E-Box region of the Snail binding site (M) (Figure 1A and 1B). In addition, instead of using the FokI domain of TALEN, we amplified the SET domain of EHMT2 for histone methylation and sub-cloned the SET domain into the TAL-effector domain. Finally, we completed the TALE-SET module (TSET) (Figure 1B). Therefore, we hypothesize that TSET may bind to the promoter region of E-cadherin; subsequently, the SET domain in TSET can dimethylate H3K9 in the E-cadherin promoter region. This process may suppress of E-cadherin expression in cancer cells and increase cell migration/invasion.

### TSET repressed E-cadherin expression in cancer cell lines

**Figure 2:**
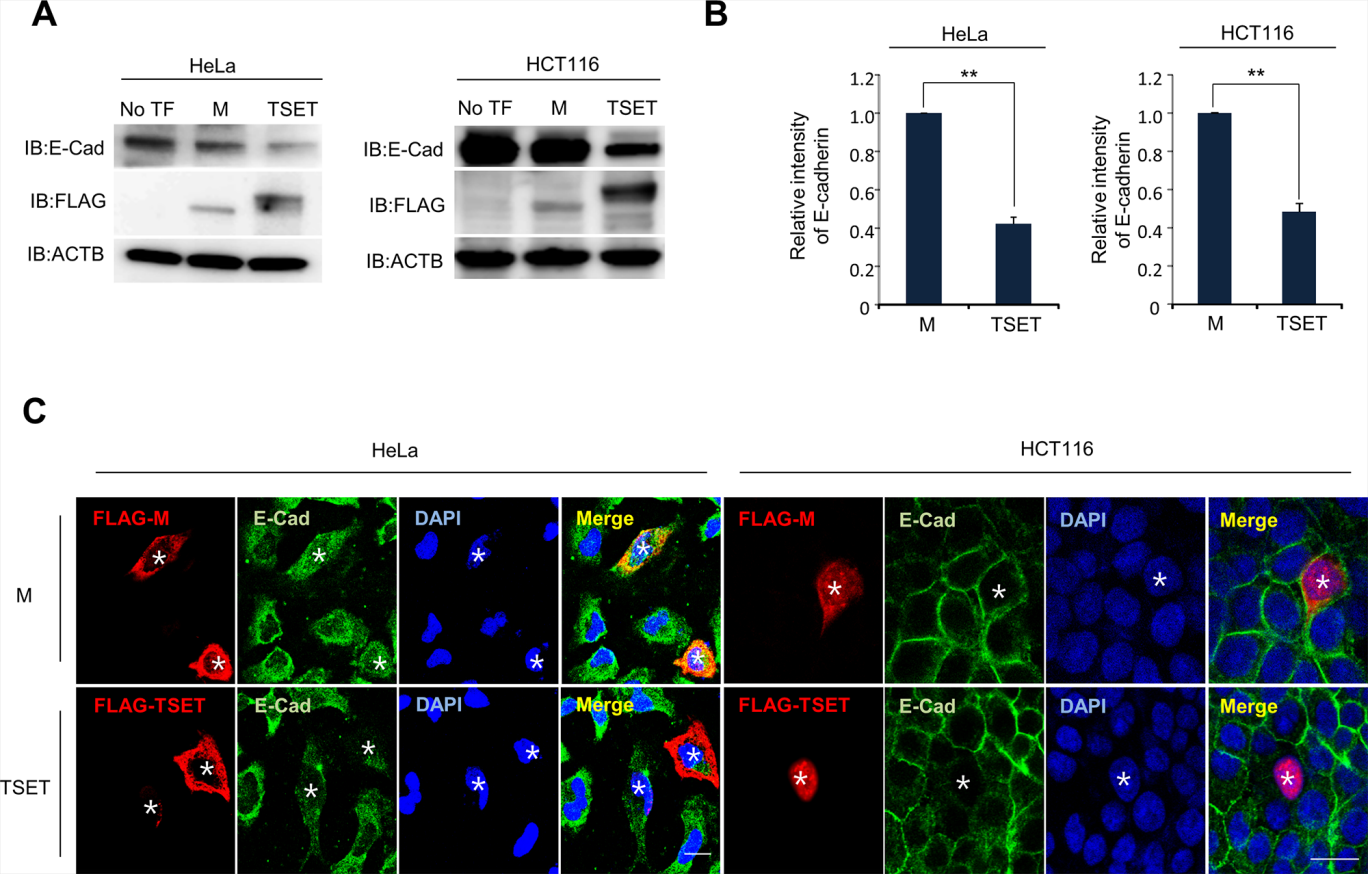
TSET represses the expression of E-cadherin in cancer cells **A.** Expression levels of E-cadherin in HeLa and HCT116 cells. FLAG-M and FLAG-TSET vectors were transfected into HeLa and HCT116 cells for 48 h, respectively. Western blot was performed to measure E-cadherin expression level, and anti-ACTB antibody was used as an internal control (No TF: No Transfection). **B.** Quantitative real-time PCR analysis showing suppression of E-cadherin expression by TSET. Relative mRNA expression shows the value normalized to the expression level of ACTB (*P* values were calculated using Student's *t* test **p < 0.01). **C.** Immunocytochemistry showed that the expression of E-cadherin was repressed by TSET. After transfection of FLAG-TSET or FLAG-M into HeLa and HCT116 cells, the cells were fixed with methanol. The fixed cells were stained with anti-E-cadherin (Alexa Fluor 488 [green]) or anti-FLAG (Alexa Fluor 594 [red]) antibodies and DAPI (blue) (*: Transfected Cell).

To assess the function of TSET, we transfected it into HeLa and HCT116 cell lines with FLAG-TSET or FLAG-M and performed western blot and quantitative RT-PCR analysis (Figure 2A and 2B). Western blot analysis showed a significant decrease in E-cadherin expression in the cells transfected with TSET compared to those transfected with No TF and M (Figure 2A). Furthermore, expression of E-cadherin was repressed at the transcriptional level by overexpression of TSET (Figure 2B). To confirm this result, we performed an immunocytochemical analysis and observed that overexpression of TSET reduced E-cadherin expression in HeLa and HCT116 cells. However, M overexpression did not change the level of E-cadherin expression (Figure 2C).

### TSET increased cell migration and invasion in cancer cells

**Figure 3:**
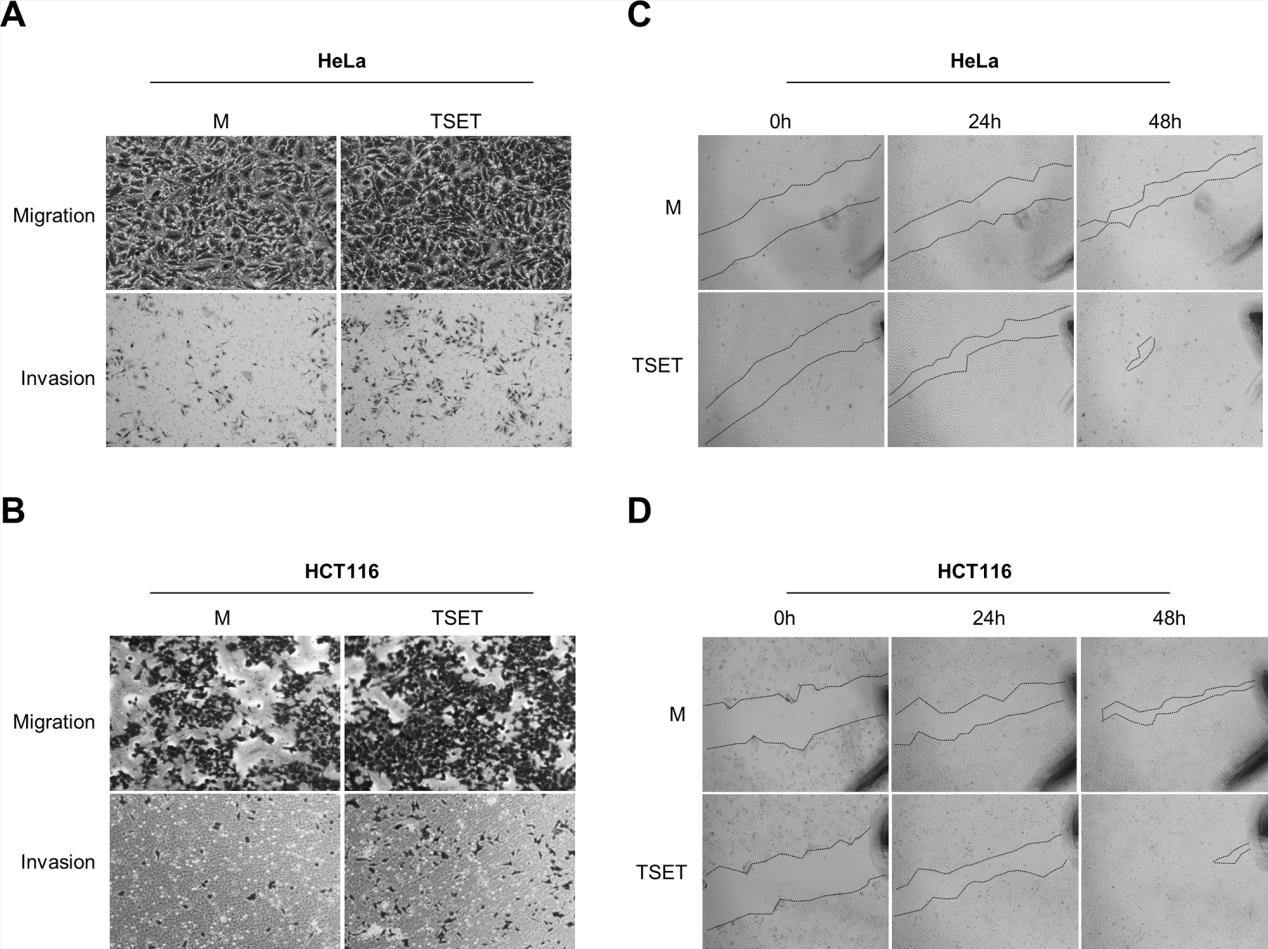
TSET increased cell migration and invasion in cancer cells **A.** and **B.** Cell migration and invasion assay in HCT116 and HeLa cells. Cells transfected with FLAG-M or FLAG-TSET were loaded into Transwell inserts and invasion chambers, respectively. Migrated and invasive cells were fixed with methanol and stained with crystal violet. **C.** and **D.** Wound healing assay in HCT116 and HeLa cells. After transfection with FLAG-M or FLAG-TSET for 24 h, wound closure was monitored with a light microscope at 0 h, 24 h, and 48 h after wound formation.

EMT processes are essential during cancer cell metastasis. EMT-activated cells have stem cell-like features and increased cell migration resulting in tumor metastasis [11–13]. We attempted to clarify the significance of E-cadherin suppression by TSET in HeLa and HCT116 cells. A cell migration, invasion and wound healing assay, showed an increase in migrated and invasive cells after transfection with TSET compared to transfection with M in HeLa and HCT116 cells (Figure 3A and 3B). We also observed high speed wound closure in TSET transfected cells (Figure 3C and 3D). These data indicate that repression of E-cadherin expression by epigenetic regulation of TSET induces cell migration and invasion in cancer cells.

### TSET enhanced H3K9 dimethylation status in the promoter region of E-cadherin

**Figure 4:**
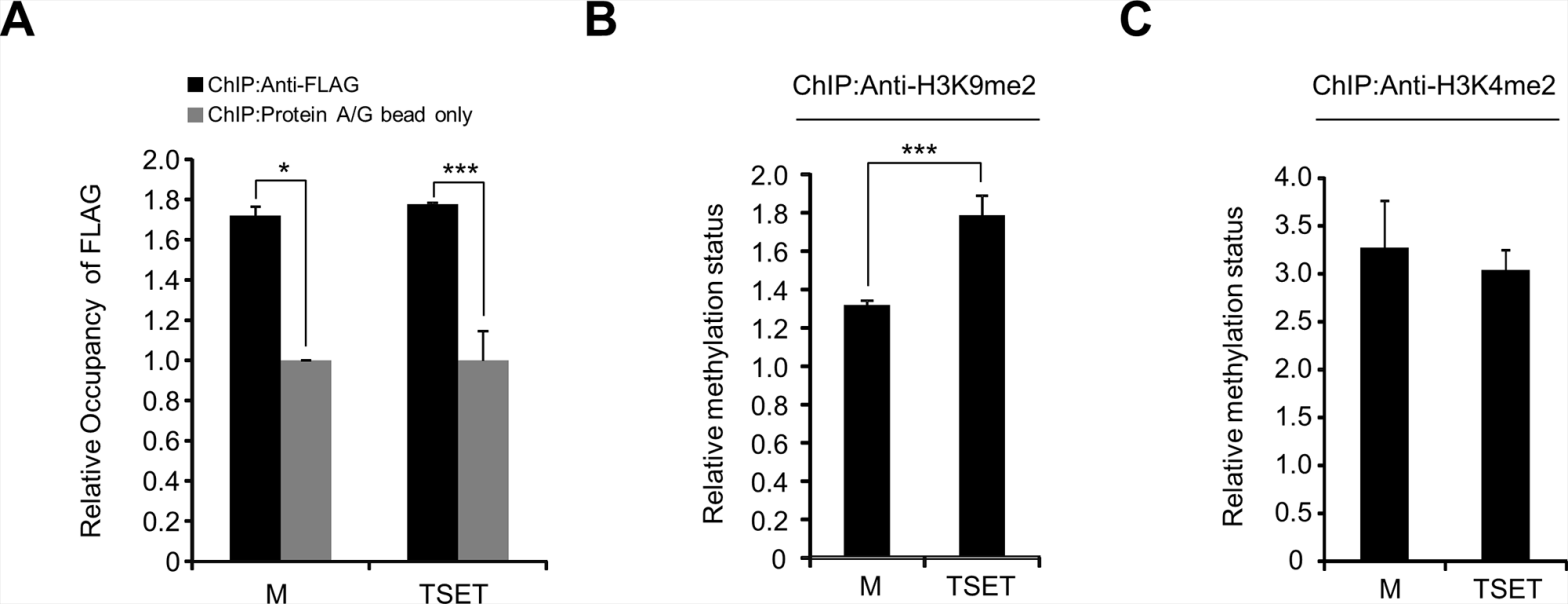
The expression of E-cadherin was directly decreased by TSET **A.** A ChIP assay was performed using anti-FLAG antibody and protein A/G bead as the negative control after HeLa cells were transfected with FLAG-M and FLAG-TSET, respectively. The results are shown as relative occupancy of the input chromatin (*P* values were calculated using Student's *t* test *p < 0.05, ***p < 0.001). **B.** and **C.** ChIP assay using anti-H3K9me2 and anti-H3K4me2 antibodies. The results are shown as relative methylation status of the input chromatin (*P* values were calculated using Student's *t* test ***p < 0.001).

To test whether TSET epigenetically regulates E-cadherin expression, we performed a ChIP assay after transfection of HeLa cells with TSET or M. Figure 4A shows that, TSET and M were enriched at the promoter region of E-cadherin in the cells transfected with TSET and M compared to the negative control (Protein A/G bead). Moreover, to assess the status of H3K9me2 in this promoter region, we performed an additional ChIP analysis with an anti-H3K9me2 antibody. The results showed that transfection with TSET increased H3K9 dimethylation compared to transfection with M (Figure 4B). However, the ChIP analysis with H3K4 dimethylation as the negative control did not reveal any difference in H3K4 dimethylation status in the TSET and M transfected cells (Figure 4C). Taken together, TSET can bind to the promoter region of E-cadherin and specifically increase dimethylation of histone H3K9, and epigenetic regulation by TSET represses E-cadherin expression in cancer cells.

## DISCUSSION

TALEs from *Xanthomonas* are transcriptional activators that specifically bind to DNA and regulate plant genes, and TALENs have site-specific endonuclease activity for selective genome cleavage [23, 24]. In the development of the TALEN system, researchers easily obtained targeted gene knockout mice [6]. Instead of using endonucleases, epigenetically active proteins, such as TET1 and LSD1, were combined with TALEs, and these new TALE-TET1 or LSD1 modules regulated targeted gene expression via site-specific DNA and histone demethylation [9, 10]. In general, to regulate gene expression at the transcriptional level, a complex network of DNA methylation and histone modification is necessary [25]. Thus, we focused on a histone methyltransferase as a new TALE-fusion protein for site-specific histone methylation (Figure 1). To assess the effects of targeted histone methylation by TSET, we selected E-cadherin as a target gene, and cell migration as a target phenotype in cancer cells. The rationale was that *Dong et al*. demonstrated the ability of histone methyltransferase G9a to increase H3K9 dimethylation in the promoter region of E-cadherin via interaction with Snail. Moreover, the repression of E-cadherin expression by G9a induces cell migration in breast cancer cell lines [16]. Snail can bind to the E-box region and repress E-cadherin expression. Two E-box regions (E-box1 and 2) exist in the promoter region of E-cadherin (Figure 1A). Thus, in this study, we designed two TALE modules to cover E-box 1 and E-box2 and constructed two TALE-SET modules (TSET1 and TSET). However, the cells that overexpressed TSET1 did not display altered E-cadherin expression (data not shown).

To test the expression level of E-cadherin in the cells that overexpressed TSET, we performed western blot, Q-RT-PCR and immunocytochemical analysis and observed a significant decrease in E-cadherin (Figure 2). In addition, we found an increase in H3K9 dimethylation in the promoter region of E-cadherin and increased cell migration and invasion after transfection with TSET (Figures 3 and 4).

There are many types of cancer therapy, including surgery, chemotherapy, and radiation therapy. However, to reduce the side effects of cancer treatment, targeted therapy has recently received increased attention. Targeted cancer therapy is a newer form of cancer therapy that specifically attacks cancer cells but not normal cells. Therefore, targeted cancer therapy is a growing component of cancer treatment [26]. To overcome side effects for cancer treatments, TALE systems are able an alternative for the treatment of cancer or other diseases because they can be quickly engineered to bind to any desired DNA sequence to control specific gene expression or editing. For example, TALE systems can control overexpression of cancer-related genes by binding to promoter regions or enhancer regions, which can be edited by TALEN. Moreover, a fusion protein with a TAL effector domain and several histone modification domains, such as methylation, demethylation and phosphorylation domains, directly regulates targeted gene expression for cancer treatment without side effects. Therefore, this study showed that targeted histone methylation by TSET has the potential to be valuable for cancer therapy.

**Figure 5:**
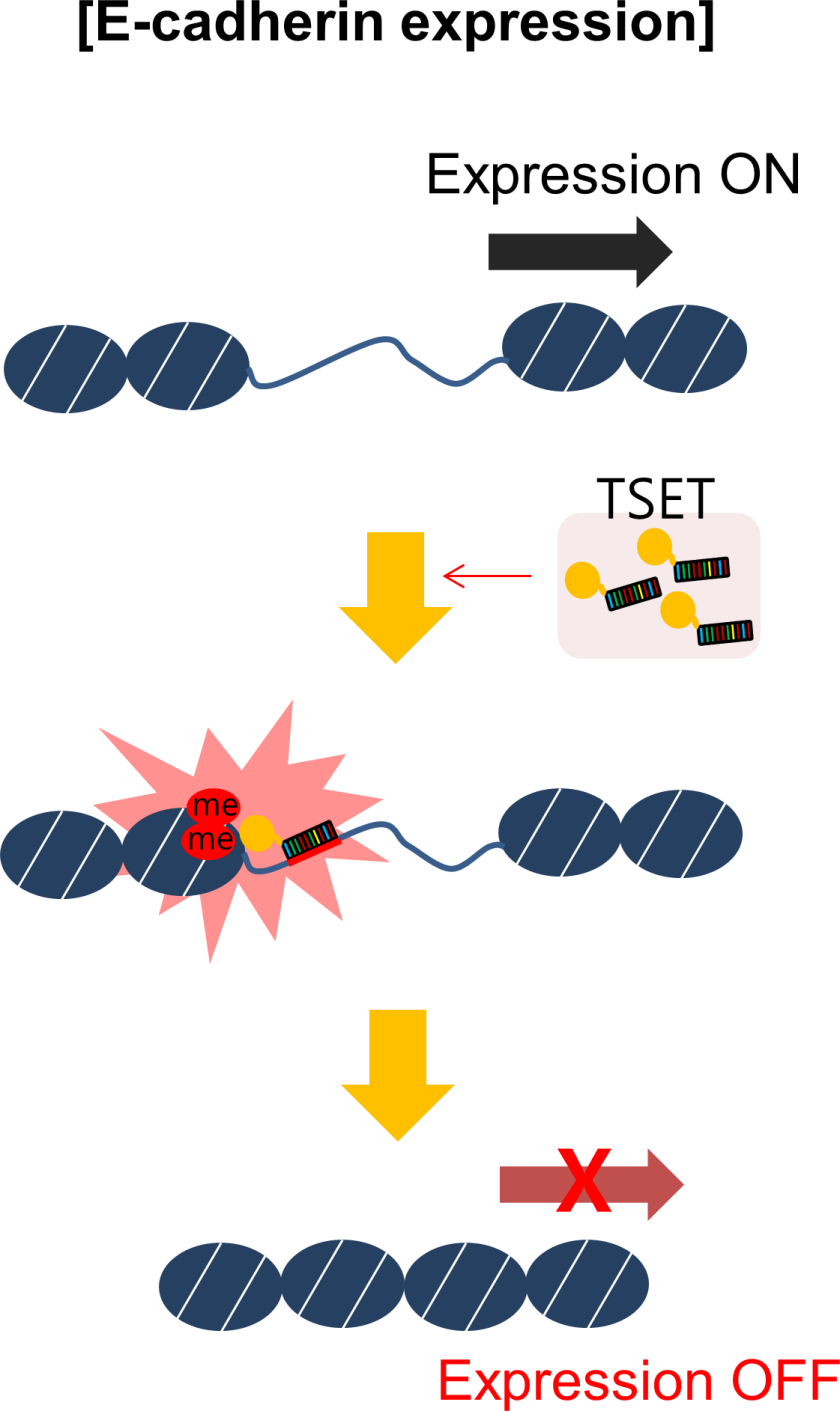
A schematic model of targeted E-cadherin regulation by the TSET system Overexpression of TSET decreased the expression of E-cadherin through an epigenetic mechanism and increased cancer cell migration and invasion.

In conclusion, to establish new targeted histone methylation, we constructed TSET for targeted regulation of E-cadherin. As shown in Figure 5, when TSET was overexpressed, it bound to the promoter region of E-cadherin and induced H3K9 dimethylation. Consequently, the expression of E-cadherin was repressed and cell migration/invasion were increased. This is the first report of a phenotype-based study of targeted histone methylation by the TALE module and provides further insight into targeted histone methylation and new cancer treatments.

## MATERIALS AND METHODS

### Construction of TSET fusion protein

The FokI domain of the TALE-FokI expression vector (pCS2TAL3-DD, Addgene) was replaced with the SET catalytic domain of EHMT2 using the restriction enzymes BamHI and XbaI such that SET was fused to the C-terminal end of the TAL effector. TALE repeat arrays were designed and assembled via multi-fragment cut-ligation using Golden Gate cloning [27]. These assembled DNA fragments were cloned into the TSET expression vector and the construct was verified by restriction enzyme digestion and sequencing. A 40-amino acid ((GGGS)_10_) linker was inserted between the TAL effector repeats and the SET domain.

### Reagents

Anti-H3K9me2 (ab1220) and H3K4me2 (ab7766) antibodies were purchased from Abcam (Cambridge, UK); anti-E-cadherin (sc-7870) ACTB(sc-47778) and HRP-conjugated secondary (sc-2031, sc-2004) antibodies were purchased from Santa Cruz Biotechnology (Texas, USA). Anti-FLAG (F1804) antibody was obtained from Sigma-Aldrich (St Louis, USA).

### Quantitative real-time PCR

Specific primers for human *ACTB* (Forward 5′-CAAGAGATGGCCACGGCTGCT-3′; Reverse 5′-TC CTTCTGCATCCTGTCGGCA-3′) and *E-cadherin* were designed (Forward 5′-TGCCCAGAAAATGAA AAAGG-3′; Reverse 5′-AAAATCTCCATTGGATCC TC-3′). PCR reactions were performed using the CFX96 Real-Time System (Bio-RAD) following the manufacturer's protocol [20].

### Cell culture

HeLa cells and HCT116 cells were maintained in DMEM supplemented with 10% FBS and 1% penicillin/streptomycin in a humidified atmosphere of 5% CO_2_ at 37°C.

### Cell migration and invasion assay

Transwell inserts were coated with a 2% gelatin solution and incubated at room temperature for 4 h for the migration assay. Transfected cells were counted and loaded onto the gelatin-coated Transwell inserts (353097, FALCON) and invasion chambers (354480, Corning) at a concentration of 5 × 10^4^ cells/well. The cells were incubated in a 5% CO_2_ incubator at 37°C for 24 h. Migrated and invasive cells were fixed with methanol for 5 min and stained with 0.1% crystal violet.

### Wound healing assay

A pipette tip was used to make a straight scratch to simulate a wound. Wound closure was monitored with a light microscope and imaging software at 0 h, 24 h and 48 h after wound formation.

### Transfection

Plasmid DNA vectors were added to the FuGENE 6 (E2691, Promega) transfection reagent and medium. The mixture was incubated for 20 min at room temperature and then added to growth medium and incubated for 48 h.

### Western blotting

Western blotting was performed following the manufacturer's protocol [28]. The cells were lysed in lysis buffer (50 mM Tris-HCl, pH 7.4, 150 mM NaCl, 1% Triton X-100, 0.1% SDS, 1 mM EDTA, 1 mM Na_3_VO_4_, 1 mM NaF, and 1x protease inhibitor cocktail) and centrifuged at 14, 000 × g for 20 min at 4°C. The protein samples were subjected to western blot with anti-FLAG, anti-E-cadherin or anti-ACTB antibodies at a 1:500 dilution ratio.

### Chromatin immunoprecipitation assay

Chromatin immunoprecipitation (ChIP) assays were performed using the ChIP Assay Kit (17-295, Millipore, Billerica, MA) according to the manufacturer's protocol. Briefly, the fragment of the TALE-SET1 module and chromatin complexes were immunoprecipitated with anti-FLAG or H3K9me2 antibodies 48 hours after transfection with the FLAG-M and FLAG-TSET vectors. DNA fragments bound to TALE-SET1, H3K9me2, and H3K4me2 were eluted, and quantitative real-time PCR was performed to quantify the bound DNA fragments. The primer sequences are shown in Figure 1A.

### Immunocytochemistry

Cells were fixed with cold methanol for 5 min at −20°C. The cells were covered with PBS containing 3% bovine serum albumin for 1 h at room temperature to block nonspecific hybridization and were then incubated with rabbit anti-FLAG or anti-E-cadherin at a 1:500 dilution ratio. After washing with PBS (−), the cells were stained with an Alexa Fluor 488-conjugated anti-rabbit secondary antibody (A11008, Life Technologies) or an Alexa Fluor 594-conjugated anti-mouse secondary antibody (A11004, Life Technologies) at a 1:250 dilution ratio. Nuclei were counterstained with 4′, 6′-diamidine-2′-phenylindole dihydrochloride (DAPI) (H-1200, Vector Lab. Inc).
